# 非小细胞肺癌ERCC1表达的转化研究现状

**DOI:** 10.3779/j.issn.1009-3419.2014.05.12

**Published:** 2014-05-20

**Authors:** 少华 崔, 丽岩 姜

**Affiliations:** 200030 上海，上海交通大学附属胸科医院 Shanghai Chest Hospital, Shanghai JiaoTong University, Shanghai, 200030, China

肺癌分子标志物研究是转化医学研究的热点问题，作为世界范围内常见的恶性肿瘤，肺癌严重威胁着人们的健康^[1]^。其中非小细胞肺癌（non-small cell lung cancer, NSCLC）约占肺癌总数的80%。切除修复交叉互补基因1（excision repair cross-complementation 1, ERCC1）是重要的肺癌分子标志物，NSCLC的ERCC1表达的转化研究取得较大进展。本文综述了ERCC1表达转化研究中的最新进展以及存在的问题，并试图据此提出合理化建议，为促进ERCC1早日向临床转化、指导NSCLC患者个体化治疗方案的制定提供依据。

## 1 ERCC1表达与NSCLC

ERCC1位于人类染色体19q13.2，是一种单链DNA核酸内切酶，是核苷酸切除修复（nucleotide-excision repair, NER）途径的限速酶，而NER途径在机体DNA损伤修复过程中发挥着重要作用。ERCC1表达可在一定水平上反映DNA修复能力（DNA repair capability, DRC）。机体DRC降低，使得细胞DNA损伤不能及时得到修复，可增强肺癌易感性。相反，ERCC1过表达可导致DNA-铂类加合物的修复，产生铂类耐药。目前，铂类联合三代化疗新药是晚期NSCLC患者的主要治疗方案，铂类在肿瘤细胞内水解成双氯双氨铂，然后形成DNA-铂类加合物，阻止肿瘤细胞DNA的复制，发挥其细胞毒性作用。

转化研究结果表明，ERCC1表达有作为NSCLC预后或铂类疗效预测的潜力，但结论并未达成一致，有必要开展进一步的研究来解决ERCC1表达转化研究中存在的一些问题。

## 2 ERCC1表达转化研究中存在的问题

### 2.1 ERCC1表达的测定方法

目前用于测定ERCC1表达水平的方法主要包括免疫组织化学技术（immunohistochemistry, IHC）和实时定量聚合酶链反应技术（real-time quantitative polymerase chain reaction, RT-PCR）。IHC和RT-PCR分别测定ERCC1蛋白水平和mRNA水平。IHC较为经济，能迅速检测肿瘤组织ERCC1蛋白表达，具有较广泛的适用性，但缺乏可重复性和客观性。RT-PCR是定量测定ERCC1 mRNA表达水平最敏感的方法，但该技术复杂、需组织样本量多、成本高，实验室普遍采用有一定困难。有研究证实，ERCC1 mRNA表达水平与蛋白表达水平间存在差异，造成两种检测方法结果不一致。Tepeli等^[2]^研究了91例NSCLC肿瘤标本ERCC1在DNA、mRNA和蛋白三个水平状态之间的关系，发现ERCC1 DNA和mRNA表达存在明显相关（*r*=0.662），而mRNA和蛋白表达之间没有明显相关性（*r*=-0.013）。Zheng等^[3]^检测44例早期术后NSCLC患者的肿瘤标本，发现ERCC1 mRNA表达水平和ERCC1蛋白表达水平无明显相关性。Ozdemir等^[4]^采用IHC法对83例Ⅲb期或Ⅳ期NSCLC患者治疗前肿瘤活检标本进行ERCC1蛋白水平检测，患者均接受铂类化疗，且化疗前未接受过手术或放射治疗，结果表明ERCC1蛋白表达阳性者和阴性者的化疗疗效、无进展生存期（progression-free survival, PFS）、总生存期（overall survival, OS）的差异无统计学意义。Ren等^[5]^采用RT-PCR法对100例Ⅲb期或Ⅳ期NSCLC患者ERCC1 mRNA水平进行检测，患者接受铂类和三代新药方案治疗，结果表明ERCC1 mRNA高表达者OS短。

这些结果的差异，可能与ERCC1表达测定方法不同有关。哪一种方法更适用于临床检测，还需进一步研究确定。Vilmar等^[6]^使用IHC和RT-PCR两种方法检测33例NSCLC患者手术切除的肿瘤组织包括ERCC1在内的4种基因转录和翻译水平的表达，结果表明与RT-PCR相比，IHC检测能力更强。Chen等^[7]^认为，对接受铂类化疗患者ERCC1表达水平的检测，IHC要好于RT-PCR。因为在铂类耐药机制中，*ERCC1*基因蛋白水平产物在修复DNA-铂类加合物导致的DNA损伤过程中发挥了主要作用，两种ERCC1表达检测方法的关系和适用性有待进一步研究。

需要指出的是，目前IHC测定ERCC1蛋白表达水平的研究，大多采用鼠单克隆抗体8F1，Olaussen等^[8]^近来的一项研究表明自2006年以来，某些原因可能使8F1抗体的特异性发生了改变。Ma等^[9]^通过高密度蛋白微阵列芯片技术发现目前因8F1抗体会与一种RCYT1A蛋白发生交叉反应而已经不再适用于ERCC1蛋白表达水平的检测。他们的研究同时发现了两个新产生的单克隆抗体4F9和2E12，对ERCC1蛋白特异性强，将来可能会替代8F1作为使用IHC方法检测ERCC1蛋白表达水平的特异性抗体。

Friboulet等^[10]^对ERCC1蛋白的四种亚型进行功能性检测，他们发现仅ERCC1-202蛋白亚型与DNA修复途径有关，DNA修复酶缺乏互补基因F（xeroderma pigmentosum complementation group F, XPF）的稳定性需要ERCC1-202蛋白亚型的表达，而其余三种亚型与DNA修复途径无关。通过选择性剪切，*ERCC1*基因产生4种分子量的mRNA，不同ERCC1 mRNA产生不同的ERCC1蛋白。而仅有11 kb的mRNA表达出39×10^9^分子量的蛋白质时，才表现为对铂类耐药，这种功能性蛋白亚型在肿瘤细胞DNA的NER途径中起主要作用。而目前研究使用的抗体尚无法辨别*ERCC1*基因产生的三种非功能性蛋白和一种功能性蛋白，检测得到的是ERCC1总蛋白表达水平^[8, 10]^，因此，如果我们能找到一种能够特异检测功能性ERCC1蛋白亚型的抗体，将可以通过功能性ERCC1蛋白亚型表达水平更准确地预测NSCLC患者对铂类药物的敏感性。

### 2.2 ERCC1表达高低划分点的确定

Ozdemir等^[4]^采用IHC法测定ERCC1蛋白表达，他们根据染色的范围和强度，将染色范围分为0分为无，1分为1%-9%，2分为10%-49%，3分为≥50%；染色强度分为0分为无，1分为弱，2分为中等，3分为强。计算染色强度与染色范围的比值为H评分。将所有H评分的中位数作为ERCC1表达高低或阳性/阴性标准的划分点。他们采用IHC法对83例Ⅲb期或Ⅳ期接受铂类方案治疗的NSCLC患者治疗前肿瘤活检标本进行ERCC1蛋白水平检测，结果表明ERCC1蛋白表达阳性者和阴性者的化疗疗效、PFS、OS的差异无统计学意义。Holm等^[11]^的实验同样采用IHC法，但他们将染色范围分为0分为无，0.1分为1%-9%，0.5分为10%-49%，1分为≥50%。计算H评分，并将H评分高于0者定义为ERCC1高表达。他们对163例接受顺铂和吉西他滨治疗的NSCLC患者ERCC1蛋白表达和中位OS的关系进行了评估，发现ERCC1低表达的男性患者中位OS长。Booton等^[12]^采用RT-PCR法测定ERCC1 mRNA表达水平，用入组的66例NSCLC患者ERCC1 mRNA表达水平中位数作为高低表达划分标准，他们对入组患者ERCC1 mRNA表达和铂类化疗反应、化疗毒性的关系进行评估，发现ERCC1 mRNA表达和化疗反应、毒性反应之间不存在明显关系。

Hubner等^[13]^的一项*meta*分析对23项研究的调查发现，无论研究者采用IHC法还是RT-PCR法，ERCC1表达水平高低划分标准差异较大。15项采用IHC法测定ERCC1蛋白表达水平的实验中，研究者大多采用H评分划分ERCC1高低表达水平，但标准各异。而8项采用RT-PCR法测定ERCC1 mRNA表达水平的实验中，划分标准同样不同。

目前，对于ERCC1表达水平划分标准的不一致，研究者大多根据各自实验样本划分高低表达水平，使得不同研究结果间的比较出现问题。转化研究阶段，需要统一划分标准才可能使ERCC1作为分子预后和预测标志物用于临床，因此，还需大量前瞻性分层研究来确定合适的ERCC1表达划分标准。

### 2.3 不同来源标本ERCC1表达的不同

检测ERCC1表达水平所需样本一般来源于手术切除或组织活检。但研究发现由这两种方法取得的标本ERCC1表达水平有差异。大样本和小样本ERCC1表达的不同可能造成研究结果的不一致。Taillade等^[14]^采用IHC法比较了NSCLC患者活检标本和相应手术取得全部肿瘤标本中ERCC1的表达水平，发现对于来自同一患者的两种标本ERCC1表达水平高度相关（*r*=0.83），但是并不完全一致，两种方法对ERCC1表达阳性检测仍存在9%的差异。27例标本中，3例手术切除标本ERCC1表达阳性而支气管活检结果却呈阴性。说明活检标本并不能完全取代全部肿瘤标本进行ERCC1表达水平检测。Jakobsen等^[15]^获得6例Ⅰ期和Ⅱa期接受胸腔镜切除的NSCLC腺癌患者标本，每个切除标本被分成三等份并包埋在石蜡包块中，每一包块切下4 μm厚度的部分涂在玻片上，前后两部分用于HE染色、组织学确诊和IHC分析（包括评估所有切片肿瘤组织最佳代表部位），在每个HE着色的部分标注5个直径约5 mm的圆形区域（标本中央和周围四个角），采用IHC法对每个肿瘤标本的15个部位进行包括ERCC1在内的六种蛋白表达的检测，发现对于同一种分子标志物，同一标本不同部位表达存在差异，除胸苷酸合成酶外，存在33%-87%的差异。这些差异提示依据小样本检测分子标志物表达的局限性。

对于早期发现并行手术切除的NSCLC患者，手术切除病灶可作为ERCC1检测样本。但确诊NSCLC时，大多患者已属中晚期，失去手术治疗机会，组织活检为取得样本的主要途径。但用支气管镜或经皮肺活检取得的样本量少，有些取样并不能完全代表整个肿瘤组织，而IHC、RT-PCR两种检测方法都需要高质量和足够数量的样本，这就可能造成检测到的ERCC1表达水平与真实情况之间的差异。因此，为最大可能减少由于样本量过少造成的这种差异，对于不能手术的晚期NSCLC患者，应选择重复、多点取样，以增强样本的代表性和检测的准确性。

### 2.4 原发病灶和转移病灶ERCC1表达的不同

Gomez等^[16]^采用IHC方法对49例NSCLC患者原发病灶和相应转移病灶中四种分子标记物表达进行测定，结果显示原发病灶和相应转移病灶分子标志物表达存在差异，其中20例患者（41%）原发病灶和相应转移病灶ERCC1表达水平不同。他们发现转移病灶ERCC1表达水平高于相应原发灶，特别是发生脑转移和肾上腺转移的NSCLC患者。原发病灶和转移病灶ERCC1表达水平的不同，可能是生物标志物在肿瘤本身表达的不同或在疾病进展过程中*ERCC1*基因表达谱发生了生物学改变^[2]^。

NSCLC确诊时许多患者出现了转移病灶。有无转移是判断患者预后的重要指标，如以单一原发或转移病灶ERCC1表达水平为标准评价患者预后，可能导致评价失误。而对于其中接受含铂方案治疗的患者，仅检测原发病灶ERCC1表达可能不足以准确预测铂类药物疗效，以此为指导制定晚期NSCLC发生转移患者的个体化疗方案可能无法达到预期效果。因此，对晚期NSCLC发生转移性疾病患者测定ERCC1表达病灶的选择，还需进一步探讨。

## 3 探讨其他检测ERCC1表达的可能途径

肿瘤组织多点、重复取样虽可提高ERCC1表达水平检测的准确率，但对晚期NSCLC患者造成极大痛苦，其他简便、快捷的检测方法有必要进一步研究。血液标本容易获取，通过检测循环肿瘤细胞ERCC1表达水平指导转移NSCLC患者的个体化治疗是一种值得考虑的方法。Zhang等^[17]^利用RT-PCR技术检测49例接受吉西他滨和顺铂治疗的NSCLC患者外周血和肿瘤组织中核糖核苷酸还原酶M1（ribonucleotide reductase M1, RRM1）、ERCC1 mRNA表达，评价ERCC1表达与临床和病理因素、治疗反应、患者预后的关系，结果发现两个基因表达均与临床和病理因素无关，肿瘤组织ERCC1 mRNA低表达者化疗疗效和预后较好（中位OS长），RRM1 mRNA表达在外周血与肿瘤组织中呈正相关（*r*=0.332, *P*=0.020），但两种组织中ERCC1 mRNA表达无相关性（*r*=0.258, *P*=0.073），不能预测铂类化疗效果。这提示通过外周血检测到ERCC1表达可能不能充分反映肿瘤组织ERCC1的表达。

Das等^[18]^采用一种新的循环肿瘤细胞检测技术评价17例发生远处转移且接受铂类药物治疗的NSCLC患者循环肿瘤细胞ERCC1表达与预后的关系，他们利用一种使用光纤阵列扫描技术的高速扫描仪检测循环肿瘤细胞中的分子标记物，结果表明ERCC1低表达患者的PFS长。但由于其研究样本量较少，这种方法是否可行需要通过进一步研究证实。

## 4 ERCC1表达结合其他因素指导NSCLC患者个体化治疗

### 4.1 ERCC1联合其他分子标志物检测制定NSCLC患者个体化治疗方案

肺癌是一种多因素疾病，肺癌患者肿瘤细胞中可能出现多种遗传学改变。除了通过ERCC1的检测评价患者预后和预测铂类疗效之外，同时对其他有意义的分子标志物进行检测，可大大提高对NSCLC患者制定“量体裁衣”式个体化治疗方案的准确性。Azuma等^[19]^评价ERCC1和β-微管蛋白Ⅲ的表达与45例接受卡铂和紫杉醇方案的NSCLC患者疗效的关系，发现ERCC1和β-微管蛋白Ⅲ表达均阴性者PFS与OS明显长于其他表达组合的患者。卡铂和紫杉醇是临床治疗NSCLC常用的化疗药组合，同时对可能与这两种药耐药有关的分子标志物进行检测，可更好的选择能使用该方案进行化疗的患者，使预期化疗效果达到最佳。

### 4.2 结合分子因素和临床因素共同制定NSCLC患者个体化治疗方案

目前，强调基于分子分型个体化治疗的同时，我们仍不能忽视临床因素，结合分子和临床因素共同制定的个体化治疗方案才是对“量体裁衣”最恰当的诠释。这些临床因素主要包括患者个人状况如性别、年龄、职业、吸烟情况、生活环境、家庭情况（遗传因素）、体力状态、体重减轻情况、其他与肺癌有关的呼吸系统疾病史等；以及肿瘤组织状况包括病理组织学类型、临床分期等因素。

Holm等^[11]^采用IHC法检测ERCC1表达水平对接受卡铂和吉西他滨方案治疗的不能手术的NSCLC患者的影响，他们对性别因素进行分层分析，163例入组患者中包括81例男性和82例女性。结果表明，ERCC1表达水平对患者预后的影响仅表现在男性，而ERCC1表达阳性或阴性对女性患者生存率影响的差异无统计学意义。同时，他们还发现相比腺癌，鳞癌患者更容易表现出ERCC1阳性。Lee等^[20]^研究发现ERCC1高表达与鳞癌组织学类型具有相关性。目前，大多数对ERCC1表达水平与患者预后或铂类耐药的研究中，入组患者大多为男性，实验未对性别因素进行分层分析，故没有确切得到性别因素与ERCC1表达水平是否具有一定关系，而肺癌的组织学类型是否与ERCC1表达高低有关，也需进一步研究证实。Wang等^[21]^研究发现，同样采用顺铂为主的化疗方案，Ⅰ期和Ⅱ期-Ⅲa期NSCLC患者ERCC1表达对预后的作用不同，Ⅰ期ERCC1高表达者预后好；而Ⅱ期-Ⅲa期ERCC1低表达者预后好，这表明个体化治疗方案中，肿瘤的临床分期是一个不可忽视的因素。此外，Azuma等^[22]^研究发现具有ERCC1阴性表达和体力状态较好的接受铂类药物化疗的NSCLC患者OS长。

## 5 探索基于ERCC1分子标志物的NSCLC治疗新策略

ERCC1表达水平与铂类耐药的确切关系需要进一步研究证实。然而，目前研究多是基于ERCC1表达水平来评价患者的预后和铂类药物疗效的预测作用。如果ERCC1表达水平确实与铂类疗效存在确切的相关性，那么我们可以通过抑制ERCC1表达水平来增强NSCLC高表达患者对铂类药物的敏感性，探索基于ERCC1分子标志物的NSCLC治疗新策略。Chang等^[23]^采用小干扰RNA（small interfering RNA, siRNA）技术抑制ERCC1表达水平，研究细胞对铂类药物的敏感性。结果表明，使用ERCC1 siRNA特异性抑制ERCC1表达水平可提高人类肿瘤细胞对铂类药物的敏感性，将来ERCC1 siRNA技术可能成为一种新的、高效的治疗策略用于铂类化疗方案中。

## 6 小结

转化医学研究存在的问题表明，ERCC1能否作为独立评价NSCLC患者预后与铂类药物疗效预测的分子标志物的问题仍不能得到解答。但ERCC1是具有研究价值的肺癌分子标志物，其在转化阶段存在的问题，仍有待解决。在今后的研究设计过程中，需要着重开展大样本量的前瞻性随机对照临床试验，对可能的混杂因素进行必要的控制，并且使用统一的评价指标，得出更有助于临床确定个体化治疗方案的结论，以更好的指导临床实践。
